# Progress in Medicine

**Published:** 1897-12-18

**Authors:** 


### PROGRESS IN MEDICINE.

### DISEASES OF THE LUNGS.

(*Continued from page* 187).

**Hæmoptysis**, as is well known, occurs from the lungs, even when no abnormal physical signs are to be discovered in the chest. Hence, if it is clear that the blood is not coming from the gums or mucous membrane of the mouth, and there is no constitutional malady, such as hæmophilia, purpura or scurvy, or any of the forms of anæmia, the conclusion is at once come to that the patient is threatened with phthisis. A number of cases, however, have recently been recorded by Newman,[14] in which hæmoptysis has frequently occurred in persons not suffering from phthisis, and in which the blood has come from lesions of the larynx or pharynx. The chief causes are—acute inflammation, and the various forms of ulceration of the mucous membrane, and less evident lesions, such as varicose veins; also very vascular tumours in the larynx, at the base of the tongue, or in the naso-pharynx.

In some of these cases the blood trickled down into the larynx or trachea, and was expectorated as if from the lungs. It is thought by many that if blood be seen in the trachea, we may conclude that the hæmoptysis comes from the lungs; but this is not altogether correct, for blood may flow and even collect in the trachea from the higher air-passages. It may be asserted, however, that in hæmorrhage from the larynx or parts above it the trachea is seldom blood-stained, and the blood expectorated is pure, only on the surface of a mass of mucus, inspissated or in dry clots. Whereas during pulmonary hæmoptysis the trachea is blood-stained, and the blood is intimately mixed with the mucus.

In any case, then, of hæmoptysis, in which no obvious lung disease exists, careful search should be made by laryngoscope and rhinoscope for a possible lesion in the upper air-passages.

The following treatment of hæmoptysis from phthisis is advocated by Wethered.[15] If the bleeding is profuse, no benefit is effected by giving astringents by the mouth. The best plan is absolute rest in bed, and a small dose, say one-sixth of a grain, of morphia by hypodermic injection, and a little ice to suck (not too much, for flatulent colic may be thus produced). Later, saline aperients may be given, and a milk diet.

In the treatment of slight hæmoptysis, astringents are more useful, and we may employ agents to allay cough if that be troublesome. ɱxv. of tincture of hamamelis, combined with a little dilute sulphuric acid, acts very well; ʒi to ʒii of liquid extract of hydrastes canadensis may also be tried. Stimulant remedies must be avoided, even should actual syncope occur.

Venesection was practised by Ewart[16] in a case of persistent hæmoptysis in phthisis. The pulse tension was high and not brought down by copious purgation, so, the hæmoptysis continuing, eight ounces of blood were taken from the arm, with very good results.

The dangers of hæmoptysis in phthisis are sometimes trifling, sometimes serious, and hence it is of some importance to know exactly what they are, and in what class of case serious consequences may be expected to arise. Chapman considers them under three heads: (1) From the loss of blood and nervous shock; (2) from suffocation from copious bleeding; (3) from mixed infection of other lung tissue through aspiration of blood with the contents of cavities. Death from suffocation is happily rare, and, if it occur, it is rapid, being due to rupture of some aneurism in a cavity. Hence, as Wethered says, if the patient is alive when the medical man reaches him, he will, in all probability, survive that particular attack. The real danger is from pneumonia, which may set in within a couple of days of the hæmorrhage, and which involves, it may be, a number of lobules or even an entire lobe, and may occur in both lungs. The phenomena are due almost certainly to the fact that cavities in phthisis contain other organisms—*e.g.*, streptococci—which pass with blood into the bronchi, and infect other portions of the lungs. This complication is apt to occur where there is active destructive disease going on in the lung, or when caseous pneumonia co-exists. The gravity of this complication of phthisis is borne out by Von Ruck's statistics, which show that 70 per cent. of the hæmorrhages under his observation occurred in cases without active disease or destructive processes, and in most instances could be referred to over-exertion; in 30 per cent. of these the course of the disease was adversely influenced, whilst the remaining 30 per cent. of hæmorrhages occurred during caseous pneumonia or active destructive disease, and 60 per cent. of these were adversely affected. It would appear, then, that in any cases of phthisis in which hæmoptysis occurs, though the immediate danger is over when the hæmorrhage ceases, yet one must wait a few days until the danger of pneumonia is gone by before being able to give a good prognosis *quâ* the hæmorrhage.

West[17] gives some valuable statistics as to the mortality and duration of pneumothorax, and deduces therefrom the data on which a prognosis may be made in any case. The general mortality of pneumothorax is about 70 per cent., *i.e.*, rather more than two cases out of three die. Of those who die 75 per cent. do so within the first fortnight, and 90 per cent. within the first month. The highest rate of mortality is that of the first day, and it steadily falls off as time goes on. The real mortality of pneumothorax is difficult to arrive at, for it often happens that the patient gets over the pneumothorax and yet dies before long either from the diseases which caused it or from the results to which the pneumothorax indirectly leads. Death may be directly due to the pneumothorax itself, and it is then caused by suffocation. This is in most cases brought about by the sudden embarrassment of the circulation and respiration consequent on the disease, and it is not so much the amount of change as the suddenness of it which is the cause of the grave symptoms. Every hour that the patient keeps alive the better is his chance of passing safely through the initial dangers. Another and different cause of suffocation lies in the discharge of contents of cavities into the air-tubes, which get sucked into the remaining lung and thus choke the patient. When death follows at a later period, it is often the result of the effusion consequent on the pneumothorax. These effusions are, roughly speaking, serous in one-third of the cases, sero-purulent in another third, and purulent in the remaining third. Where the effusion is purulent, the side has to be opened sooner or later, and there is consequently an open empyema under unusually unfavourable conditions. The patient dies usually from asthenia, the result partly of the prolonged discharge, and partly from the phthisis. Again, the patient may die, not from the pneumothorax, but from the original disease which produced it—which is usually phthisis. In these cases the pneumothorax is merely a grave complication which accelerates the end.

[14] Brit. Med. Jour., May 29, 1897. [15] Clinical Journal, March 17, 1897. [16] Lancet, August 28, 1897. [17] Lancet, May 8 and 15, 1897.

### FEVERS.

**Influenza.**—Fraenkel[1] discusses the complications affecting the lungs. He met with 80 cases of broncho-pneumonia out of a total of 272. There is a curious absence of fibrinous exudation, and often no rusty sputum, in the typical form, but mixed cases containing both the pneumococcus and the influenza bacillus also exist, and when an epidemic prevails ordinary pneumonia is very prevalent without any observable cause. Pulmonary gangrene is apt to follow the inflammation, and frequently goes on to pneumothorax or to a stinking empyæma, because the pleura becomes perforated. Gresswell[2] divides attacks of this disease into four classes—(1) Pulmonary; (2) abdominal; (3) neurotic; (4) rheumatic; as well as a malignant or typhoid form, and notices the sudden onset occasionally seen which may be even mistaken for apoplexy.

**Typhoid.**—The recent epidemics at Maidstone, Lynn, Clifton, and elsewhere, though probably all due to local accidents, have brought out the ever present danger of this disease. Full investigations have not yet been carried out, but at Maidstone[3] the epidemic followed closely the distribution of the water from one special supply, at any rate in the first instance. Whether this was contaminated by typhoid excreta from hop-pickers or by percolation from the manure applied to the land remains to be discovered. The Clifton outbreak was entirely a milk one, following exactly the distribution of the supply from one farm which was usually taken round a given beat. Another dealer who innocently borrowed a few cans found that a crop of cases of the disease marked that portion of his walk along which he had distributed it. The danger of consuming unsterilised milk in the face of the constant danger of accidents such as this is a lesson which few people have learnt. Lynn was attacked with a similar outbreak to its present one five years ago, and still retains a water supply derived from a small river open to contamination, and one source of which is a spring arising under Grimston Churchyard.[4] Another outbreak of 80 cases has occurred at Ligoniel, a village in the outskirts of Belfast, which presented a clearly water-borne infection. The district was well looked after, the only condition common to all the persons attacked was the same water supply, which arose in the limestone rock. It was discovered that typhoid had occurred near the source of this water, and that the excreta had found their way close down to the source, and finally the typhoid bacilli were demonstrated in the water of the source itself. In India[5] the incidence during 1895 of typhoid on the European army was again the highest recorded. No less than 1,870 cases were admitted to hospital, of which 477 were fatal. The majority occurred in Northern India, and the regiments forming the Chitral field force suffered severely, while the worst malarial districts escaped in comparative freedom. Boiling the water appeared to put a stop to it; but frequently a new outbreak followed after a short time, whether from evasion of the rule, or how, does not appear. The natives, with the exception of the Gurkhas of Nepaul, suffer much less than Europeans; whether this is due to hereditary immunity or to previous attacks in infancy is doubtful.

**Widal's Test.**—Shattuck[6] reported on 145 cases in the Boston City Hospital. In 125 typhoids, the reaction failed in only one; in 19 other diseases it was absent. The tests were made with liquid blood, and one hour was allowed for the reaction, which may, in some cases, be obtained towards the end of the first week of the disease, and occasionally for as long as four months. H. A. Abbott, of Philadelphia, found the reaction in 66 out of 68 cases diagnosed otherwise as typhoid. Janeway mentioned an instance where no reaction was given on the twenty-eighth day, but was obtained later. A. T. Turner,[7] at Brisbane, found a reaction in 14 cases which were proved to be typhoid, but none in 14 of other diseases which were sent to him for diagnosis. One convalescent from typhoid also showed no reaction, and the disease of a patient who gave a doubtful reaction could not be decided clinically. W. C. C. Pakes[8] gives, in tabular form, the results of 60 cases tested by serum, diluted to 5 and 50 per cent. The reactions were taken at the end of half an hour, and again at the end of twenty-four hours. He employs a special form of pipette to avoid accidents and contamination. In 20 cases of typhoid all gave the reaction with weak serum within half an hour, though one, indeed, failed, whose illness was only at the end of the first week. Two cases reacted in which typhoid had occurred six years ago, but there was none in those persons who had never suffered from it. The use of the stronger serum and taking the results after twenty-four hours were found to be untrustworthy proceedings. Indeed, it is clear that normal serum, when but slightly diluted and acting for a long period, can cause a marked reaction. On the whole, he regards the test as of great value when the result is positive, the dilution considerable, and the time allowed short. A number of negative results throughout the course of the illness are also trustworthy. Results, though of less delicacy, can be obtained without a microscope. W. H. Welch, of Baltimore,[9] takes as a time limit two hours, and prefers a large dilution (1 in 40 or 50), as no other disease has hitherto given a reaction when the serum is only 1 in 50. Wyatt Johnson, in 600 samples, half of which were typhoid, had only one case of typhoid in which no reaction was shown. R. C. Cabot obtained a positive result in 96 out of 101 cases supposed to be typhoid. In 301 of other diseases it was absent in 300, the positive case being one of pernicious anæmia in a negro. Of cases reported by others, he found that 1,740 gave a reaction out of 1,826 supposed typhoid, or 95·2 per cent., while a negative result was obtained in 96·5 per cent. out of 1,649 instances of other diseases. Kneass obtained 43 positive results in 45 typhoids in 20 minutes, while none was found in 58 of other diseases. Bloch, at Johns Hopkins, had 6·5 per cent. of failure in 46 typhoids, and some positive results in coma from tuberculosis and malaria. Biggs, of New York, uses serum diluted to 1 in 10, and takes for the time 15 minutes. J. H. Musser and Swan got a positive reaction in 34 out of 35 cases supposed to be typhoid, but none in other diseases. The examination of the stools by Elsner's method is sometimes of value as supplementing the serum method, though the latter is applicable at an earlier stage, according to Mark Richardson. Another confirmatory test, besides that of the diazo reaction, is afforded by the absence of leucocytosis in true typhoid. It is curious that blood from the liver or spleen, as well as the bile, gives little or no reaction, while horses' blood will cause clumping in both typhoid and colon bacilli. Fœtal blood gives sometimes positive and at others negative results, when the mothers are suffering from typhoid.

Da Costa[10] reports four positive reactions out of 116 persons examined who suffered from other diseases. One case was a valvular heart disease, two were pneumonias, one of which had possibly suffered from typhoid previously, and one was neurotic œdema. In the blood of 25 normal persons no reaction was given. He obtained 95 reactions in 102 cases of typhoid, one failure being due to testing on the fourth day only of the illness. In five, however, the results were not seen for more than an hour. It should be added that he employed dried blood only. Adelaide Peckham[11] is said to have found that, while some cultures of typhoid bacilli fail to react with typhoid serum, certain growths of the bacillus coli do give a positive result. But the writer who refers to this fact neglects to state the qualifying details as to time, dilution, and the other tests relied on, though he claims in a similar manner to have obtained a reaction himself from typhoid growths tested with serum from appendicitis, and refers to Malvoz as having caused clumping by strong poisons, and even by weak solutions of crysoidine and resorcin.

[1] B. Med. Jour., Sept. 18. [2] Lancet, Sept. 18. [3] Lancet, Oct. 2. [4] Lancet, Nov. 6. [5] Lancet, June 26. [6] Med. Rec., May 8. [7] Australas. Med. Gaz., May 20. [8] Lancet, May 29. [9] Med. Rec., July 3. [10] N. Y. Med. Jour., Aug. 21. [11] N. Y. Med. Jour., Oct. 2.

